# Interaction between elevated temperature and different types of Na-salicylate treatment in *Brachypodium dystachion*

**DOI:** 10.1371/journal.pone.0227608

**Published:** 2020-01-13

**Authors:** Tibor Janda, Magdalena Anna Lejmel, Anna Borbála Molnár, Imre Majláth, Magda Pál, Quang Trung Nguyen, Ngoc Tung Nguyen, Van Nhan Le, Gabriella Szalai

**Affiliations:** 1 Centre for Agricultural Research, Agricultural Institute, Hungarian Academy of Sciences, Martonvásár, Hungary; 2 Center for Research and Technology Transfer, Vietnam Academy of Science and Technology, Hanoi City, Vietnam; 3 Institute of Research and Development, Duy Tan University, Da Nang, Vietnam; Pacific Northwest National Laboratory, UNITED STATES

## Abstract

Salicylic acid (SA) plays a role in several physiological processes in plants. Exogenously applied SA is a promising tool to reduce stress sensitivity. However, the mode of action may depend on how the treatment was performed and environmental conditions may alter the effects of SA. In the present study the physiological and biochemical effects of different modes of application (soaking seeds prior sowing; spraying leaves with 0.5 mM NaSA) were compared at normal and moderately elevated temperatures (4 h; 35°C) in *Brachypodium distachyon* (L.) P. Beauv. plants. While soaking the seeds stimulated plant growth, spraying caused mild stress, as indicated by the chlorophyll-a fluorescence induction parameters and changes in certain protective compounds, such as glutathione, flavonoids or antioxidant enzymes. Elevated temperature also caused an increase in the glutathione-S-transferase activity, and this increase was more pronounced in plants pre-treated with NaSA. Both seed soaking or spraying with NaSA and exposure to heat treatment at 35°C reduced the abscisic acid levels in the leaves. In contrast to abscisic acid, the jasmonic acid level in the leaves were increased by both spraying and heat treatment. The present results suggest that different modes of application may induce different physiological processes, after which plants respond differently to heat treatment. Since these results were obtained with a model plants, further experiments are required to clarify how these changes occur in crop plants, especially in cereals.

## Introduction

Salicylic acid (SA) serves as a signal molecule, and may induce various responses under biotic and abiotic stress conditions [1–5]. Exogenously applied SA can also be effectively used to improve the stress tolerance of various plant species. SA has been applied in different ways, for example by spraying the leaves, irrigating the plants with SA [6–8], or soaking seeds in SA solution before sowing [9–12]. The transport of endogenous SA has been characterised at several levels, such as in short- and long-distance transport processes [13], although changes in various biochemical and physiological stress acclimation processes have been described in SA-treated plants, the uptake mechanism, mode of action and further fate of exogenously applied SA is still not fully understood. For example, the cuticle may serve as a major barrier preventing the free diffusion of SA into the plant [13, 14]. The SA taken up by pea seeds from hydroponic solution was converted to conjugated forms, which newly synthesised SA was detected in other organs [15]. The majority of reports on the effects of exogenous SA do not differentiate between the different application modes, and very few compare them. It was recently demonstrated that different SA treatments, such as hydroponic treatment and seed soaking induce different physiological and biochemical processes in wheat plants, suggesting that specific results on the application of exogenous SA cannot be generalized in order to reveal the mode of action of SA [16].

High temperature is one of the most frequent abiotic stress factors. Due to global warming, the adaptation of plants to elevated temperatures is an increasingly serious problem worldwide. The application of exogenous salicylic acid has been demonstrated as a potential tool to provide protection against heat injury in plants [17]. The mechanisms proposed to explain the mode of action of SA against heat injury include the induction of antioxidant capacity [17], changes in proline production and ethylene formation [18], and the regulation of Ca^2+^ homeostasis [19]. SA may also induce genes encoding chaperone or, heat shock proteins, protein kinases, or various types of secondary metabolites [16, 20,21].

Besides the acid form of SA, other related compounds, such as acetyl-SA or the sodium salt NaSA, could also be effectively used to improve stress tolerance [2, 22,23]. These compounds have several advantages, including their better water solubility. It was recently shown that SA and NaSA alleviated Cd toxicity to different extents in maize plants [2]. The main aims of the present study were: i. to compare the physiological and biochemical effects of various types of NaSA treatment (soaking the seeds prior to sowing or spraying the leaves with NaSA) in the monocot model plant *Brachypodium dystachion*; ii. to describe how these plants respond to short-term temperature elevation, and to investigate how elevated temperature modifies the responses of *Brachypodium* to different NaSA treatments. To achieve these goals the present work mainly focused on certain antioxidant mechanisms and the synthesis of phenolic compounds.

## Materials and methods

### Plant material

*Brachypodium* seeds (*Brachypodium distachyon* (L.) P. Beauv. Bd21 (obtained from the USDA-ARS National Plant Germplasm Inventory *Brachypodium distachyon* Collection) were soaked overnight either in distilled water (control; “DW” plants) or in 0.5 mM NaSA (NaSA seed-soaked; “SS” plants). The seeds were then sown in boxes containing 3:1 (v:v) loamy soil and sand (8 boxes for DW and 4 boxes for SS plants, each box contained 39 plants). The plants were grown for 5 weeks at 20/18°C with 16/8-h light/dark periodicity and photosynthetic photon flux density (PPFD) of 250 μmol m^-2^ s^-1^ in a Conviron PGR-15 plant growth chamber (Controlled Environments Ltd, Winnipeg, Canada) in the phytotron of the Agricultural Institute, Centre for Agricultural Research, Hungarian Academy of Sciences, Martonvásár, Hungary. At the end of this period the leaves of half of the DW plants (4 boxes) were sprayed with 0.5 mM NaSA (“Spray” plants) and after one day of spraying 2 boxes from each treatment (DW, SS, Spray) was used as 20°C control. Rest of the plants (2 boxes from each treatment) were exposed to elevated temperature (35 °C) for 4 hours (S1 Fig). Photosynthetic parameters (n = 7) and shoot length and weight (at least 30) were measured, and leaf samples were collected before and after the temperature treatment (n = 5 for each biochemical analysis). Plants can be seen after 35°C 4h in S2 Fig.

### Chlorophyll-*a* fluorescence induction analysis (FI)

FI analysis was carried out on the third fully developed leaves in both temperature treatments using a pulse amplitude modulated (PAM) fluorometer with a blue LED-Array Illumination Unit [IMAG-MAX/L (λ = 450 nm)] (Imaging-PAM MSeries, Walz, Effeltrich, Germany). The plants were dark-adapted for 15 min before the first saturation pulse (SP) involving PPFD = 3000 μmol m^-2^ s^-1^ and 0.8 sec duration. Quenching analysis was carried out using actinic light with PPFD = 250 μmol m^-2^ s^-1^ and 30 sec saturation pulse frequency provided by the blue LED lamp. The Fv/Fm parameter was determined after the first SP. The steady state quantum yield and quenching parameters [effective PSII quantum yield Y(II), quantum yield of regulated energy dissipation Y(NPQ), quantum yield of non-regulated energy dissipation Y(NO), non-photochemical quenching (NPQ)] were analysed after a 15-min quenching period, as described by Klughammer and Schreiber [24].

### Estimation of lipid peroxidation

Lipid peroxidation analysis was based on the measurement of the malondialdehyde (MDA) level in 0.2 g plant leaves according to Thomas et al. [25]. The MDA concentration was measured spectrophotometrically at 532 nm, with the subtraction of non-specific absorption at 600 nm. The concentration of lipid peroxides, together with the oxidatively modified proteins, was then quantified in terms of the MDA level using an extinction coefficient of 155 mM^-1^ cm^-1^, and expressed as nM g^-1^ fresh weight.

### Antioxidant enzyme assays

For the measurement of antioxidant enzyme activity, 0.5 g of leaves were homogenized in 2.5 mL of ice-cold Tris buffer (0.5 M, pH 7.5) containing 3 mM MgCl_2_ and 1 mM EDTA. The catalase (CAT; EC 1.11.1.6) activity of the extract was monitored spectrophotometrically as the decrease in absorbance at 240 nm [26]. The ascorbate peroxidase (APX; EC 1.11.1.11) activity was measured in the presence of 0.2 M Tris buffer (pH 7.8) and 5.625 mM ascorbic acid. The reaction was started with 0.042% H_2_O_2_ and the decrease in absorbance at 290 nm was monitored. The guaiacol peroxidase (POD; EC 1.11.1.7) activity was determined at 470 nm as described by Ádám et al. [26]. The glutathione reductase (GR; EC 1.6.4.2) activity was monitored at 412 nm according to Smith et al. [27]. The glutathione-S-transferase (GST; EC 2.5.1.18) activity was measured by monitoring changes in the absorbance at 340 nm using the method of Mannervik and Guthenberg [28]. The activities were expressed in nkatal g^-1^ fresh weight.

### Extraction and analytical procedure of salicylic acid and flavonols

Flavonoids, jasmonic acid (JA), abscisic acid (ABA), SA and its precursors were measured according to Meuwly and Métraux [29] and Pál et al. [30] using 1 g plant material. Just before the HPLC analysis, the evaporated samples were resuspended in 500 μl 15% acetonitrile and filtered through a 0.45 m pore size membrane filter.

SA and JA were quantified fluorimetrically using a W474 scanning fluorescence detector (Waters, USA). The determination of benzoic acid (BA), cinnamic acid (CA), ABA and flavonols, namely rutin, myricetin, quercetin and kaempferol, was performed by means of UV spectrophotometry in the range of 210 to 320 nm (W996 photodiode array detector, Waters, USA). The parameters of the analysis can be found in S3 Fig.

### Measurement of thiols

The plant material was ground with liquid nitrogen in a mortar, after which 1 ml of 0.1 M HCl was added to 200 mg plant sample. Total glutathione content was determined after reduction with dithiothreitol and derivatisation with monobromobimane. [31]. The parameters of the analysis can be found in S3 Fig.

### Statistical analysis

The experiments were repeated four times and representative data are shown. The results were the means of five measurements. In the case of shoot length and shoot weight, data were statistically evaluated using the standard deviation and t-test methods at p<0.05 level. The effect of SA treatments under optimal and high temperatures was tested by one-way ANOVA (p<0.05) with the post-hoc Tukey’s HSD test using the Agricolae package in R environment (R v.3.5.2).

## Results

### Effects of NaSA treatments on physiological parameters in *Brachypodium*

**Fig 1:**
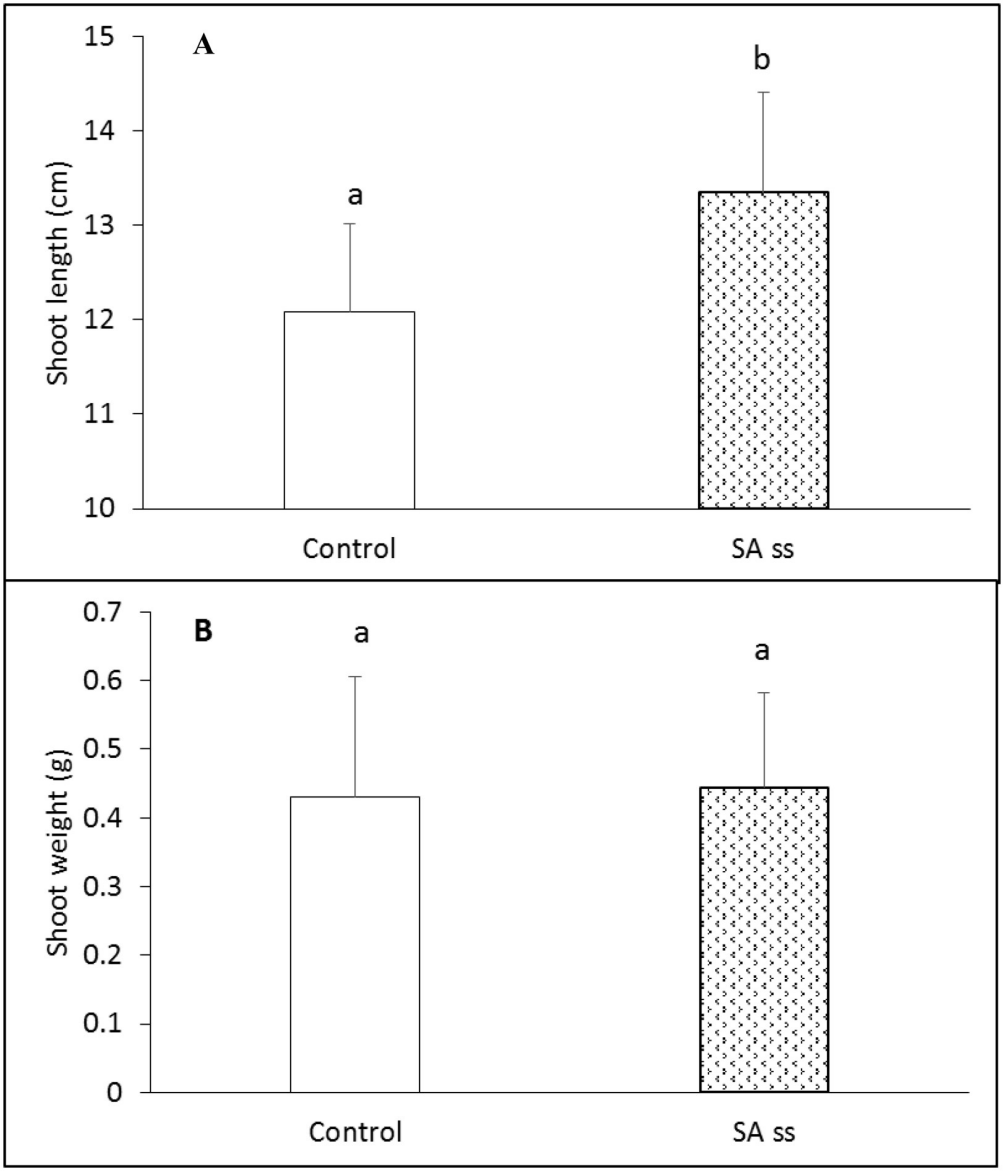
Shoot length (A) and weight (B) of 5-week-old control and seed-soaked (SS) *Brachypodium* plants grown at 20/18°C. Data represent the means of at least 25 samples with SD. The different letters indicate statistically significant differences at the p<0.05 level.

Soaking the seeds in 0.5 mM NaSA before sowing (SS) caused a slight but statistically significant increase in the shoot length (Fig 1A) but not in the shoot weight (Fig 1B) after 5 weeks growth at 20/18°C. After 5 weeks half of the control plants (DW) were sprayed with NaSA (Spray) solution and after a further day half of the plants from each group were heat-treated at 35°C for 4 h. Chlorophyll-a fluorescence parameters were determined to characterise the physiological status of plants (Table 1). The Fv/Fm parameter representing the maximum quantum efficiency of Photosystem II (PSII) was not affected either by NaSA or heat treatment, suggesting that none of the treatments caused severe stress. The actual quantum yield of PSII increased slightly after the heat treatment in the DW and SS plants, while spraying caused a slight but not significant decrease in this parameter. In contrast, Y(NPQ) decreased slightly in both DW and SS plants. The changes in the non-regulated NPQ ((Y(NO)) were not statistically significant in any of the treatments. These results suggest that, in contrast to spraying with 0.5 mM NaSA, short heat treatment at 35°C was stimulative rather than stressful.

**Table 1:** Chlorophyll-a fluorescence induction parameters in plants grown from seeds soaked in distilled water (DW) or NaSA seed-soaked (SS) or sprayed with 0.5 mM NaSA, and before (20°C) and after 4 h heat treatment at 35°C. Mean values ± SD; n = 5. The different letters indicate statistically significant differences at the p<0.05 level.

	Fv/Fm	Y(II)	Y(NPQ)	Y(NO)
**DW**				
20°C	0.796±0.005**a**	0.257±0.025**b**	0.563±0.017**a**	0.180±0.009**a**
35°C	0.794±0.002**a**	0.313±0.023**a**	0.502±0.021**b**	0.185±0.005**a**
**Sprayed**				
20°C	0.795±0.006**a**	0.224±0.029**b**	0.593±0.025**a**	0.183±0.009**a**
35°C	0.800±0.006**a**	0.242±0.025**b**	0.574±0.030**a**	0.184±0.006**a**
**SS**				
20°C	0.798±0.002**a**	0.239±0.015**b**	0.576±0.011**a**	0.185±0.004**a**
35°C	0.787±0.012**a**	0.342±0.038**a**	0.468±0.035**b**	0.190±0.004**a**

### Changes in salicylic acid contents

**Fig 2:**
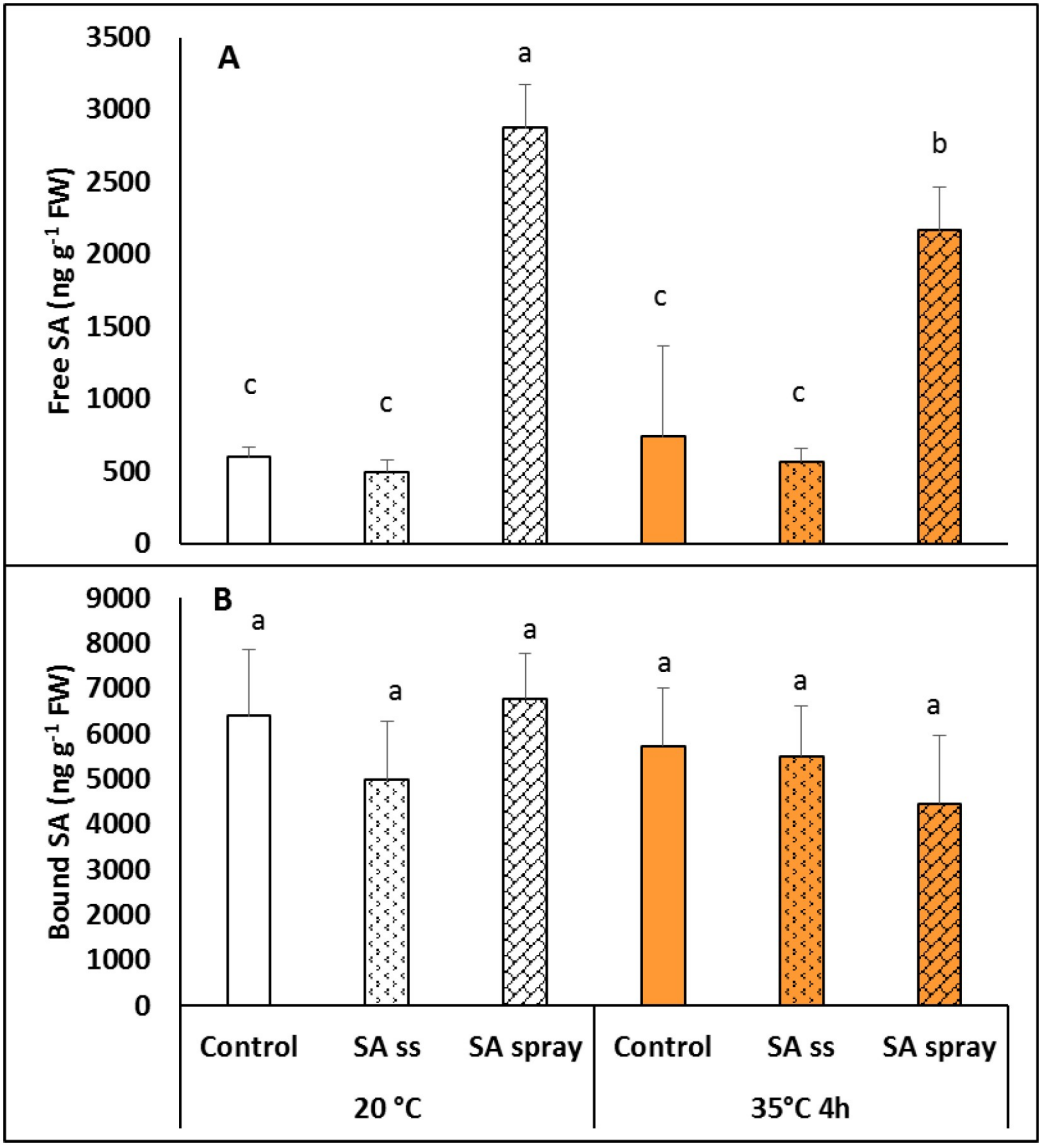
Free (A) and bound (B) salicylic acid levels in plants grown from seeds soaked in distilled water (DW) or NaSA seed-soaked (SS) or sprayed with 0.5 mM NaSA, and before (20°C) and after 4 h heat treatment at 35°C. Mean values ± SD; n = 5. The different letters indicate statistically significant differences at the p<0.05 level.

Neither pre-soaking the seeds in NaSA solution or exposure to 35°C for 4 h significantly increased the free SA content in the leaves. However, as expected, very high SA levels were detected in the sprayed leaves, which were slightly lower after the heat treatment (Fig 2A). The bound SA content did not change during the treatments (Fig 2B).

**Fig 3:**
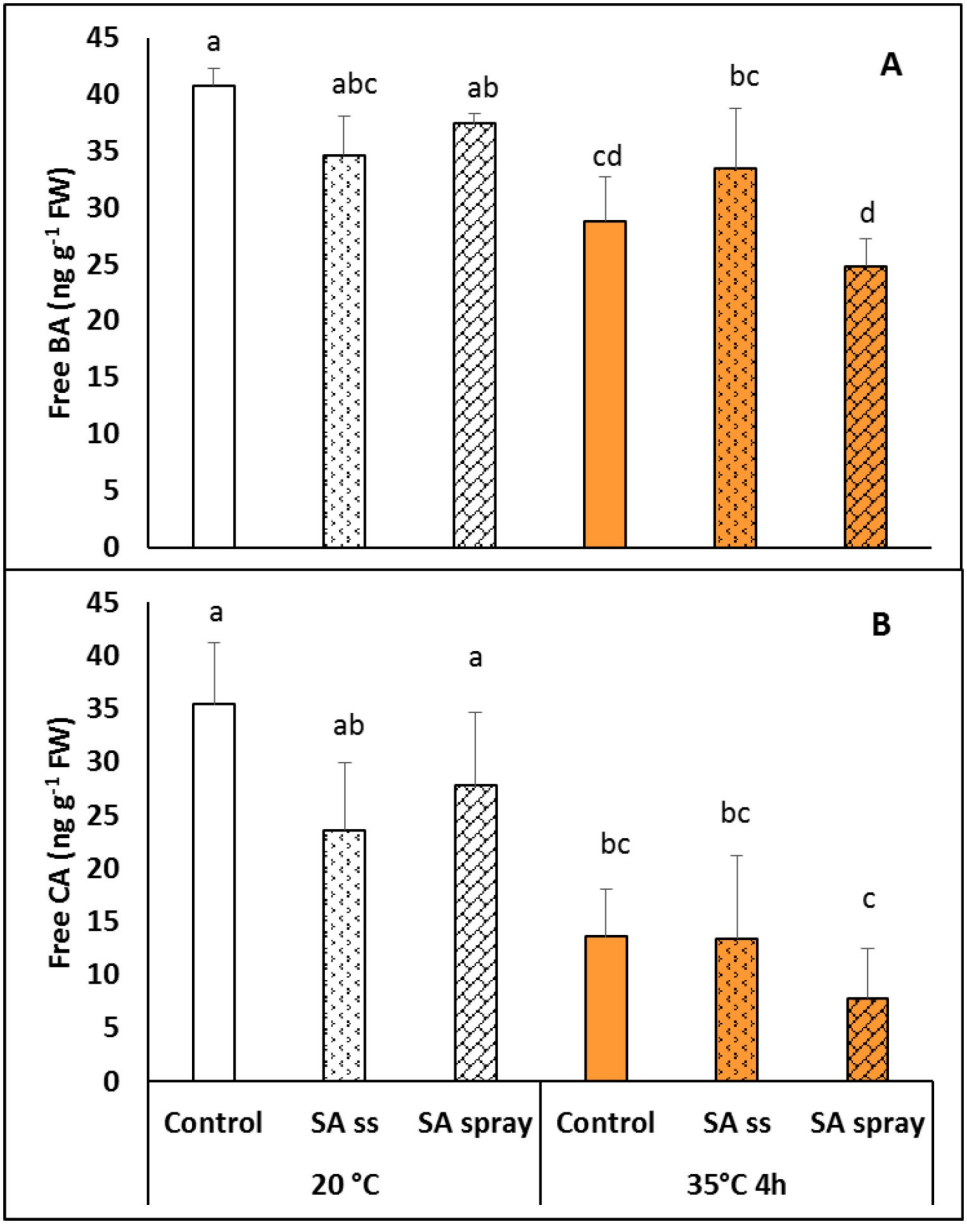
Benzoic (A) and cinnamic (B) acid levels in plants grown from seeds soaked in distilled water (DW) or NaSA seed-soaked (SS) or sprayed with 0.5 mM NaSA, and before (20°C) and after 4 h heat treatment at 35°C. Mean values ± SD; n = 5. The different letters indicate statistically significant differences at the p<0.05 level.

Some of the possible precursors of SA were also measured. The free benzoic acid (BA) content at 20°C was slightly, but not significantly lower in plants pre-treated with NaSA than in DW plants. Exposing the plants to 35°C for 4 h decreased the BA levels in both DW and sprayed *Brachypodium* plants (Fig 3A). Similar changes were found in the level of free cinnamic acid (CA), wich was lower in SS than in DW plants, and was reduced by heat treatment (Fig 3B).

### Antioxidant activity in NaSA-treated plants before and after heat treatment

**Fig 4:**
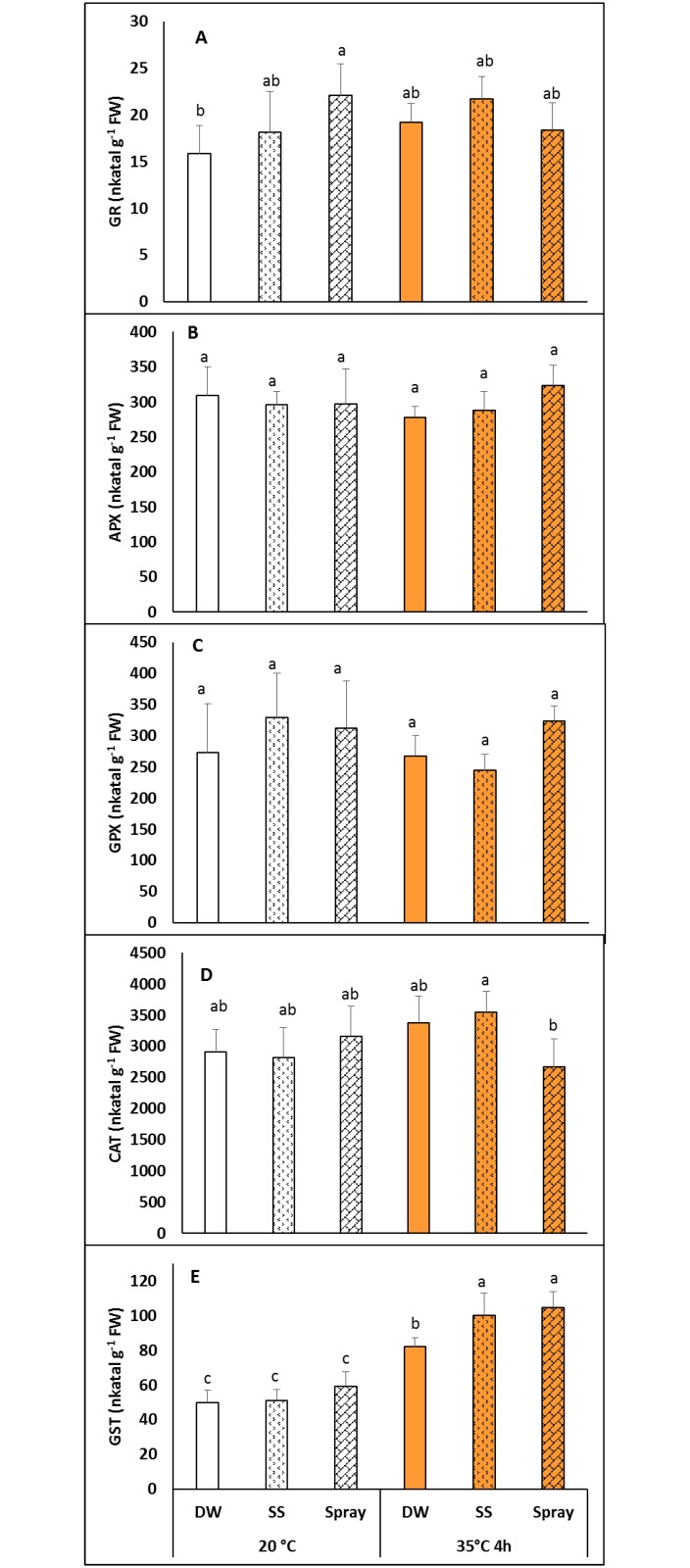
Changes in the activity of antioxidant enzymes in plants grown from seeds soaked in distilled water (DW) or NaSA seed-soaked (SS) or sprayed with 0.5 mM NaSA, and before (20°C) and after 4 h heat treatment at 35°C. A: glutathione reductase; B: ascorbate peroxidase; C: guaiacol peroxidase; D: catalase; E: glutathione-S-transferase. Mean values ± SD; n = 5. The different letters indicate statistically significant differences at the p<0.05 level.

The activity of antioxidant enzymes did not usually change substantially after NaSA treatment at 20°C. A significant increase in the glutathione reductase (GR) activity compared to that of DW plants was only found in the leaves of plants sprayed with NaSA (Fig 4A). The ascorbate peroxidase (APX) and guaiacol peroxidase (GPX) activities did not change during the treatments (Fig 4B and 4C). The catalase activity was slightly higher in the SS plants at 35°C than in heat-treated sprayed plants (Fig 4D). Heat treatment caused the most pronounced changes in the glutathione-S-transferase (GST) activity, it increasing it in all the treatments, but especially in plants pre-treated with NaSA either as seed-soaking or spraying which had higher GST activity than DW plants after 4 h at 35°C (Fig 4E).

**Fig 5:**
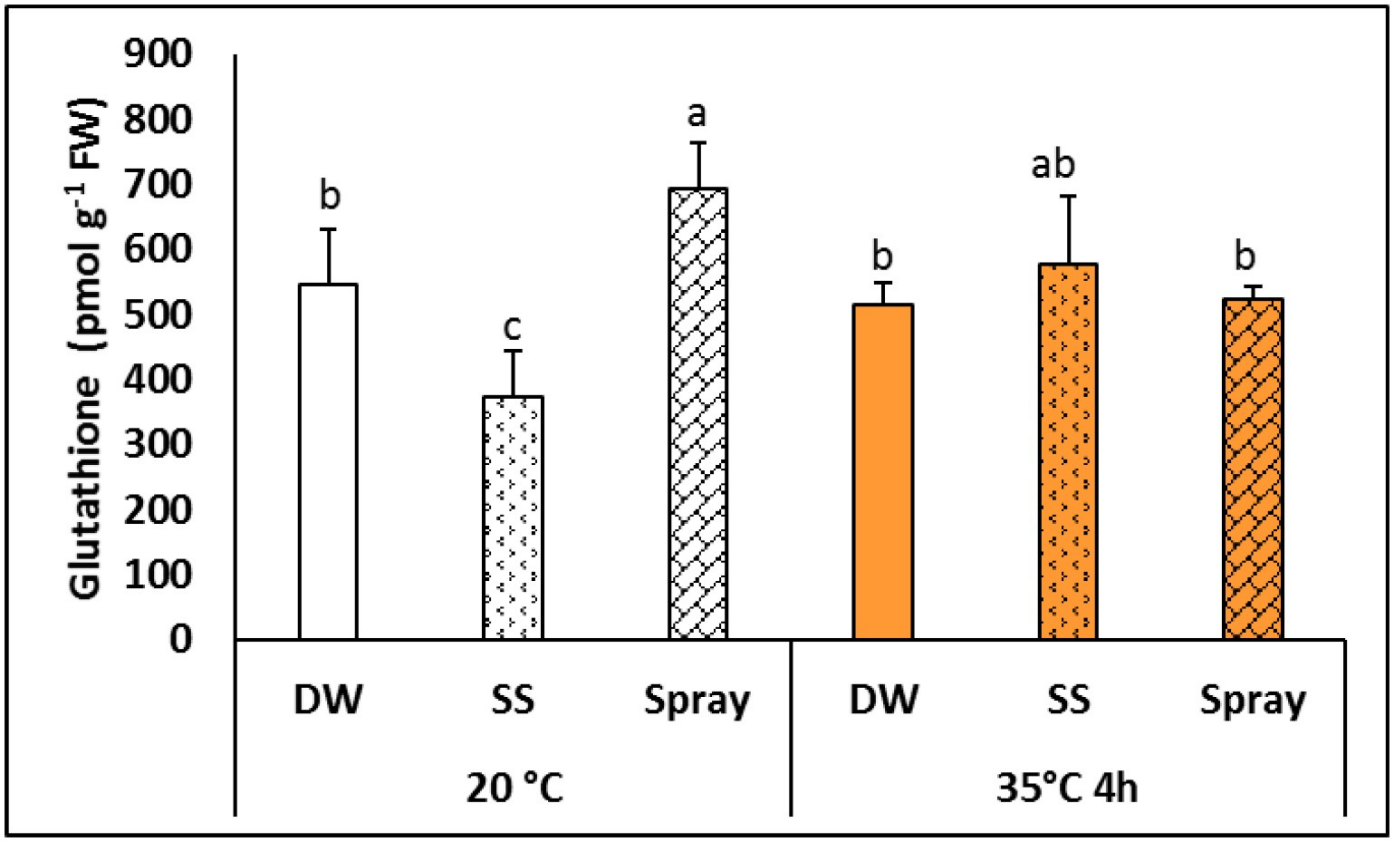
Total glutathione level in plants grown from seeds soaked in distilled water (DW) or NaSA seed-soaked (SS) or sprayed with 0.5 mM NaSA, and before (20°C) and after 4 h heat treatment at 35°C. Mean values ± SD; n = 5. The different letters indicate statistically significant differences at the p<0.05 level.

Different NaSA pre-treatments caused diverse changes in the total glutathione level, which was slightly lower in SS and higher in sprayed plants than in the controls at 20°C (Fig 5). However, these differences could not be observed after heat treatment. The amounts of the glutathione precursors cysteine, γ-glutamylcysteine and hydroxymethylglutathione and of its degradation product cysteinylglycine were also determined, but these were only influenced slightly if at all by the treatments used in this work.

### Phenolic compounds

**Fig 6:**
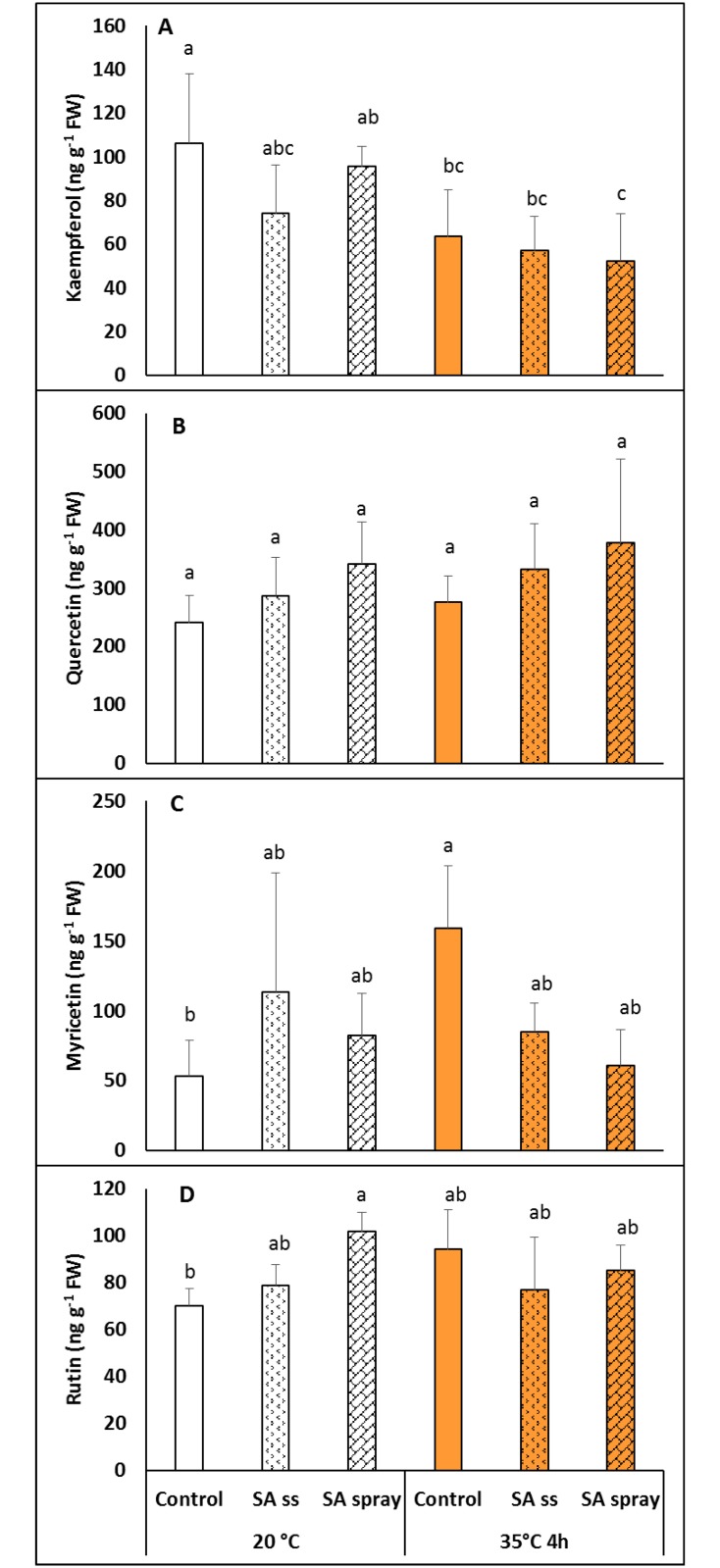
Changes in the level of various flavonols in plants grown from seeds soaked in distilled water (DW) or NaSA seed-soaked (SS) or sprayed with 0.5 mM NaSA, and before (20°C) and after 4 h heat treatment at 35°C. Mean values ± SD; n = 5. A: kaempferol; B: quercetin; C: myricetin; D: rutin. The different letters indicate statistically significant differences at the p<0.05 level.

SA is a phenolic compound, so a number of similar compounds were also investigated. Pre-treatment with NaSA at 20°C only caused an increase in rutin in sprayed plants (Fig 6). Heat treatment reduced the level of kaempferol in DW and sprayed plants, while increaseing the myricetin level in DW but not in NaSA pre-treated plants (Fig 6).

### Plant hormones (ABA and JA)

**Fig 7:**
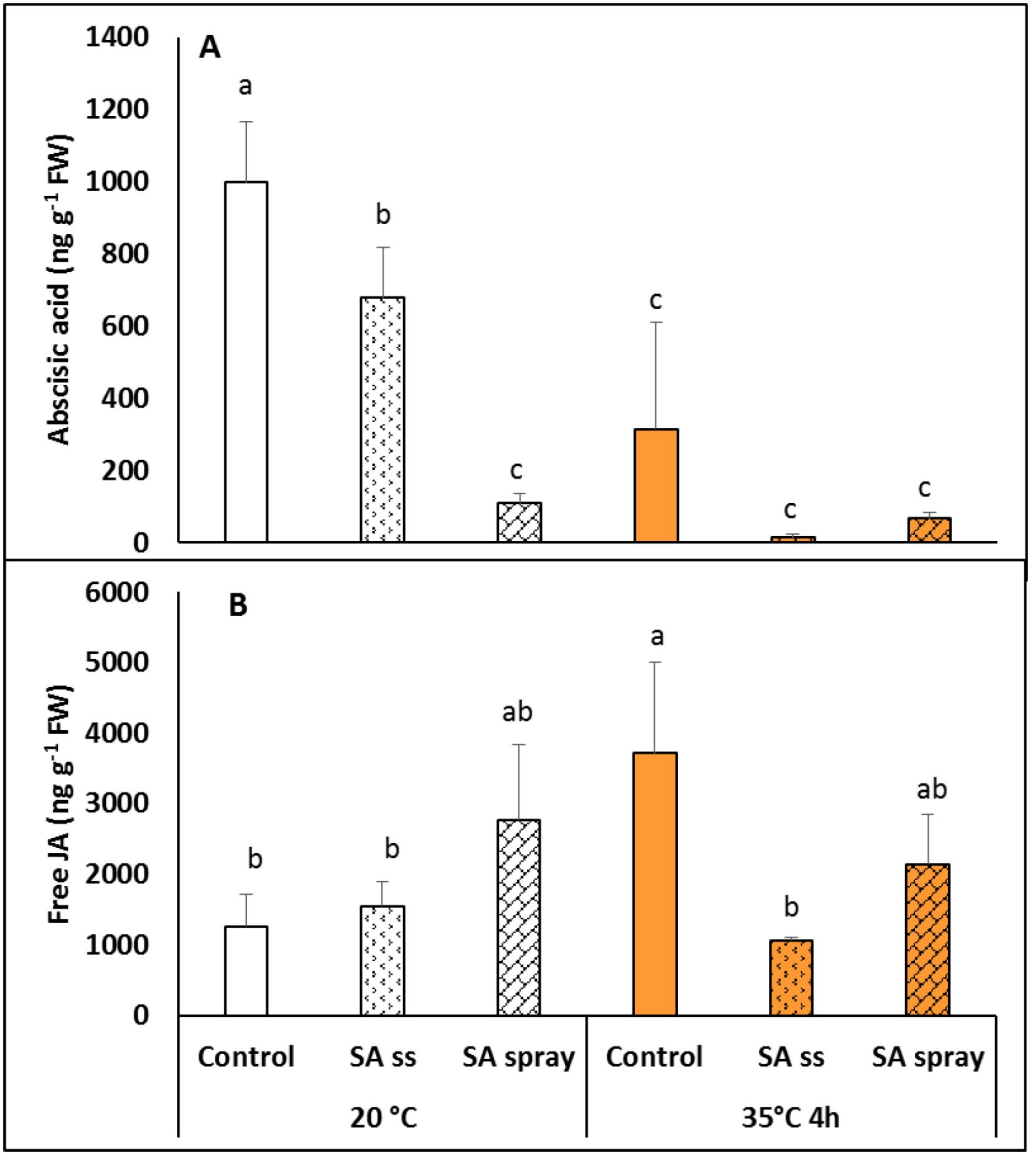
Abscisic (A) and jasmonic (B) acid levels in plants grown from seeds soaked in distilled water (DW) or NaSA seed-soaked (SS) or sprayed with 0.5 mM NaSA, and before (20°C) and after 4 h heat treatment at 35°C. Mean values ± SD; n = 5. The different letters indicate statistically significant differences at the p<0.05 level.

Pre-treatment of *Brachypodium* plants with NaSA significantly reduced the abscisic acid (ABA) level in the leaves, especially after spraying (Fig 7A). A short, 4-h heat treatment at 35°C also substantially reduced the ABA contents of DW and SS plants. Due to the pronounced reduction in ABA after heat in DW plants, the differences between the DW and NaSA-treated plants were not significant after heat-treatment. In contrast to ABA, the jasmonic acid (JA) level increased in the leaves of DW plants during heat treatment at 35°C but did not change in the other cases (Fig 7B).

## Discussion

It was recently shown that the induction of flavonoid biosynthesis pathways in wheat plants varied with the mode of SA treatment [16]. The present work involved an analogous experiment on the monocot model plant *Brachypodium dystachion* with another important abiotic stressor, the heat, while the acidic form of SA was replaced by 0.5 mM NaSA. This concentration was chosen because it had been used in several earlier studies on the effects of exogenous SA [2, 16, 22, 32]. Its effect may differ from that of SA, as recently shown in the case of Cd stress in maize plants [2]. Further research will be needed to clarify how a low concentration of Na^+^ ion interacts with SA, and whethter their effects are modified when they are applied together. Under the present experimental conditions soaking the seeds in 0.5 mM NaSA before sowing was not stressful for the plants. It increased shoot length and caused no decrease in the maximum quantum efficiency of PSII. Other studies have also shown that SA may have a positive effect on plant growth, especially when applied as seed soaking agent [33]. In some cases the positive effects of seed pre-treatment were manifested as higher grain yields, as shown for maize plants [12]. It should also be mentioned that the positive and negative effects of SA treatment depend not only on the concentration and/or mode of application but also on other environmental factors. For example, hydroponic treatment of young maize plants with 0.5 mM SA (the concentration used in the present experiment) or with related compounds such as benzoic acid or aspirin was stressful under normal growth conditions, but provided protection against low temperature stress in maize [32, 34].

The heat treatment applied also had a moderately stimulating effect. It did not reduce Fv/Fm, but, interestingly, slightly increased the actual quantum yield of PSII. However, spraying the leaves with 0.5 mM NaSA caused a slight decrease in this parameter. It is well known that SA may also cause stress, manifested as a reduction in photosynthetic processes [35]. Similarly to the actual quantum yield, changes in Y(NPQ) also indicated that both SS and mild heat treatment were mainly stimulatory, while spraying was more stressful for the plants. Exogenous SA treatment may either increase or decrease the photosynthetic activity of plants [35]. However, earlier thermoluminescence studies suggested that the effect of SA on PSII is mainly indirect [36].

The modification of antioxidant activities is one of the best known effects of SA signalling in plants [37]. One possible effect of SA is a reduction in catalase activity, which has been observed in various plant species [17, 31, 38]. In the present work NaSA treatment did not alter the activity of antioxidant enzymes in *Brachypodium* plants at 20°C, with the exception of an increase in GR activity in plants sprayed with NaSA. Increased catalase activity was only observed in NaSA-sprayed plants after exposure to elevated temperature. Similar increases in GR activity induced by exogenous SA or related compounds have also been observed in other plant species [17, 31, 34, 39]. In contrast a higher expression level of the gene encoding the GR gene has been reported in NahG *Arabidopsis* plants, which have a lower SA level, than in the wild type Col-0 plants [40]. These results show that the effects of exogenous and endogenous SA cannot be generalised, especially for different plant species. The slight changes in antioxidant enzyme activity suggested that the treatments applied did not represent severe stress for the plants. The non-enzymatic antioxidant glutathione has been reported to play a role in plant defence and acclimation processes [41], and its involvement in SA-mediated processes has also been demonstrated [42]. Although the changes detected in total glutathione levels in the present work were moderate, differences could be observed between the effects of seed soaking and spraying with NaSA. Under normal growth conditions the glutathione level was lower after seed soaking and higher after spraying than the control (DW) plants. Furthermore, elevated temperature caused a significant increase in GST activity, and this increase was more pronounced in plants pre-treated with NaSA, either as seed soaking or as spraying, than in the control. GSTs play a crucial role in various physiological processes [43], including acclimation to environmental stresses [44].

Although it has been demonstrated several times that SA plays a crucial role during plant growth and development and especially in stress responses [3, 45], its transport system is still largely unknown. Although various approaches have provided information on the intracellular and long-distance translocation of SA within cells or whole plants [13, 46,47] the mechanism(s) by which exogenous SA is taken up and how exactly it acts as a signalling molecule are poorly understood. In the present work only spraying with NaSA solution caused a significant increase in the free SA content in the leaves, but it was not possible to differentiate between the SA adsorbed on the leaf surface and that which penetrated into the cells. Neither seed soaking nor short-term heat treatment increased the SA level in the leaves. It seems that the SA level is tightly regulated by the plants. Exposure to SA regulates the whole phenylpropanoid pathway in plants, which includes the regulation of SA synthesis/metabolism and/or compartmentalisation. One mechanism by which plants cope with elevated SA levels is its conjugation in the bound form. Experiments with radio-labelled SA showed that pea plants grown from seeds soaked in SA solution can store exogenous SA in bound form in the seeds [15]. In the present experiment no increase in the bound form could be detected. When a few putative precursors were measured, decreases were observed in all the NaSA-treated plants in the levels of BA and CA (which is also a precursor of flavonols), especially in plants exposed to short-term heat treatment. This could be part of the regulation process, because BA may have an effect similar to that of SA [34].

Flavonoids are among the most bioactive secondary metabolites in plants, having substantial antioxidant activity [48]. Certain flavonoid compounds were investigated in the present work, because the accumulation of flavonols might also contribute to heat stress tolerance, as demonstrated in tomato plants [49]. It was previously shown that various kinds of SA treatment differentially modified the expression levels of genes involved in the flavonoid metabolism and the amounts of certain non-enzymatic antioxidant compounds, including the flavonol quercetin, in wheat [16]. Under the present experimental conditions the effects of seed soaking and spraying with NaSA did not differ substantially except that at 20°C only spraying significantly increased the rutin and quercetin levels in *Brachypodium* plants. Furthermore, both seed soaking and leaf spraying alleviated the heat-induced increase in the myricetin content.

In order to obtain a better understanding of SA-related signalling processes, two plant hormones, ABA and JA, were also determined. The results showed that both pre-treatment of *Brachypodium* plants with NaSA and exposure to heat treatment at 35°C reduced the ABA level in the leaves. A heat-induced decrease in ABA was also reported in *Arabidopsis* plants after at least 1 h heat treatment. Shorter-term exposure may cause a temporary increase in ABA, as reported in various plant species [50,51]. One possible explanation for the reduced ABA levels in the leaves during heat treatment is that plants tend to cool their tissues by reducing the ABA level, which is a regulator of stomatal closure [51]. It seems that exogenous NaSA could stimulate a similar response. In contrast, both spraying and heat treatment increased the JA level in the leaves. This is not surprising, because JA has been shown to be a signal transducer in heat shock, and also has a role in sesquiterpene formation [52].

In summary, although the heat stress applied in the present experiment was not severe, the results demonstrated the early responses on an hourly scale of plants to elevated temperature. The present results suggest that different application modes may induce different physiological processes, and that plants respond differently to heat treatment.

## Supporting information

S1 Fig Plant growth and treatment parameters. *Brachypodium distachyon* seeds were soaked overnight either in distilled water or in 0.5 mM NaSA. The seeds were then sown in boxes (8 boxes for DW and 4 boxes for SS plants, each box contained 39 plants). The plants were grown for 5 weeks in a plant growth chamber. At the end of this period the leaves of half of the DW plants (4 boxes) were sprayed with 0.5 mM NaSA and after one day of spraying 2–2 boxes from each treatment (DW, NaSA seed-soaked, Sprayed) was used as 20°C control. Rest of the plants (2–2 boxes from each treatment) were exposed to elevated temperature (35 °C) for 4 hours.
(PDF)

S2 Fig *Brachypodium distachyon* plants after 35°C elevated temperature treatment. (PDF)

S3 Fig HPLC analyses. A) Salicylic acid, benzoic acid, cinnamic acid, abscisic acid, jasmonic acid and flavonols, B) Glutathione.
(PDF)
