# Supplementary material for: Interaction between elevated temperature and different types of Na-salicylate treatment in Brachypodium dystachion
Source: PLoS One. 2020 Jan 13;15(1):e0227608. doi: 10.1371/journal.pone.0227608 (PMC6957344; doi:10.1371/journal.pone.0227608)

Fig S1. Plant growth and treatment parameters. *Brachypodium distachyon* seeds were soaked overnight either in distilled water or in 0.5 mM NaSA. The seeds were then sown in boxes (8 boxes for DW and 4 boxes for SS plants, each box contained 39 plants). The plants were grown for 5 weeks in a plant growth chamber. At the end of this period the leaves of half of the DW plants (4 boxes) were sprayed with 0.5 mM NaSA and after one day of spraying 2-2 boxes from each treatment (DW, NaSA seed-soaked, Sprayed) was used as 20°C control. Rest of the plants (2-2 boxes from each treatment) were exposed to elevated temperature (35 °C) for 4 hours.

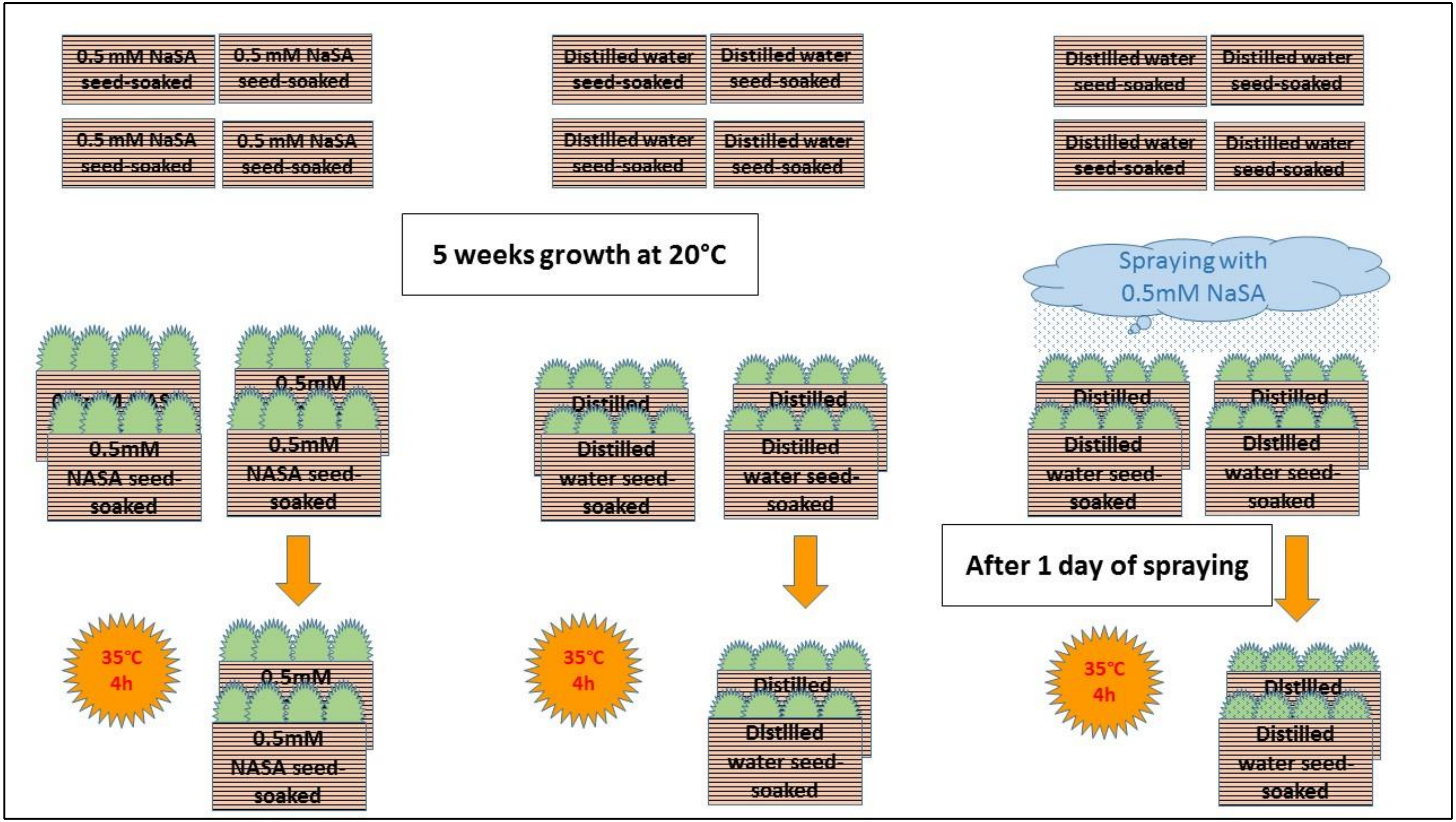

Supplement: S1 Fig — Brachypodium distachyon seeds were soaked overnight either in distilled water or in 0.5 mM NaSA. The seeds were then sown in boxes (8 boxes for DW and 4 boxes for SS plants, each box contained 39 plants). The plants were grown for 5 weeks in a plant growth chamber. At the end of this period the leaves of half of the DW plants (4 boxes) were sprayed with 0.5 mM NaSA and after one day of spraying 2–2 boxes from each treatment (DW, NaSA seed-soaked, Sprayed) was used as 20°C control. Rest of the plants (2–2 boxes from each treatment) were exposed to elevated temperature (35 °C) for 4 hours. (PDF) [file pone.0227608.s001.pdf]
