# Supplementary figures and images for: Interaction between elevated temperature and different types of Na-salicylate treatment in Brachypodium dystachion
Source: PLoS One. 2020 Jan 13;15(1):e0227608. doi: 10.1371/journal.pone.0227608 (PMC6957344; doi:10.1371/journal.pone.0227608)

**Fig S2. *Brachypodium distachyon* plants after 35°C elevated temperature treatment.**

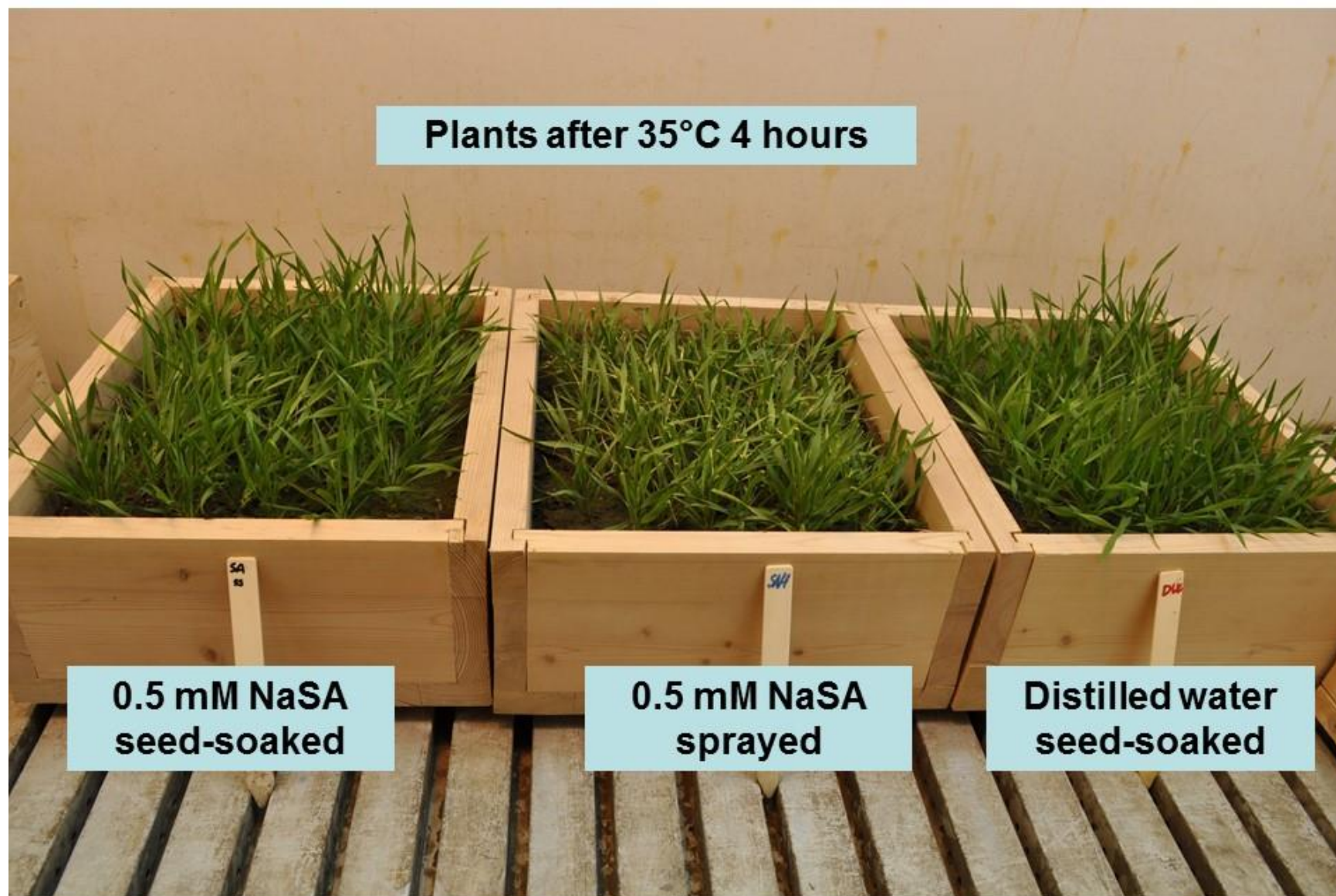

Supplement: S2 Fig — (PDF) [file pone.0227608.s002.pdf]
