# Supplementary material for: Interaction between elevated temperature and different types of Na-salicylate treatment in Brachypodium dystachion
Source: PLoS One. 2020 Jan 13;15(1):e0227608. doi: 10.1371/journal.pone.0227608 (PMC6957344; doi:10.1371/journal.pone.0227608)
Supplement: S3 Fig — A) Salicylic acid, benzoic acid, cinnamic acid, abscisic acid, jasmonic acid and flavonols, B) Glutathione. (PDF) [file pone.0227608.s003.pdf]

Fig S3. HPLC analyses

A) Salicylic acid, benzoic acid, cinnamic acid, abscisic acid, jasmonic acid and flavonols

W2690 separation modul (Waters)

Column: Synergy 4 µm Fusion-RP 80A 150x4.6 mm (Phenomenex)

Pre-column: ODS Hypersil 5 µm 10x4 mm (ThermoScientific)

Column temperature: 30 °C

Sample temperature: 10 °C

Solvents:

A: Water+0.1% HCOOH

B: Acetonitril+0.1% HCOOH

Flow rate: 1 ml min^{-1}

Gradient:

Time	A%	B%
Initial	85	15
10	50	50
11	0	100
15	0	100
16	85	15
28	85	15

Detector parameters:

W996 photodiode array detector (Waters):

Wavelength range: 210-320 nm; Sampling rate: 5 spectra/s; Resolution: 2.4 nm

Detected compounds:

240 nm: benzoic acid (RT: 8.198); cinnamic acid (RT: 9.533)

260 nm: rutin (RT: 6.014); myricetin (RT: 8.261); abscisic acid (RT: 9.181); quercetin (RT: 9.859); kaempferol (RT: 11.485)

300 nm: jasmonic acid (RT: 10.990)

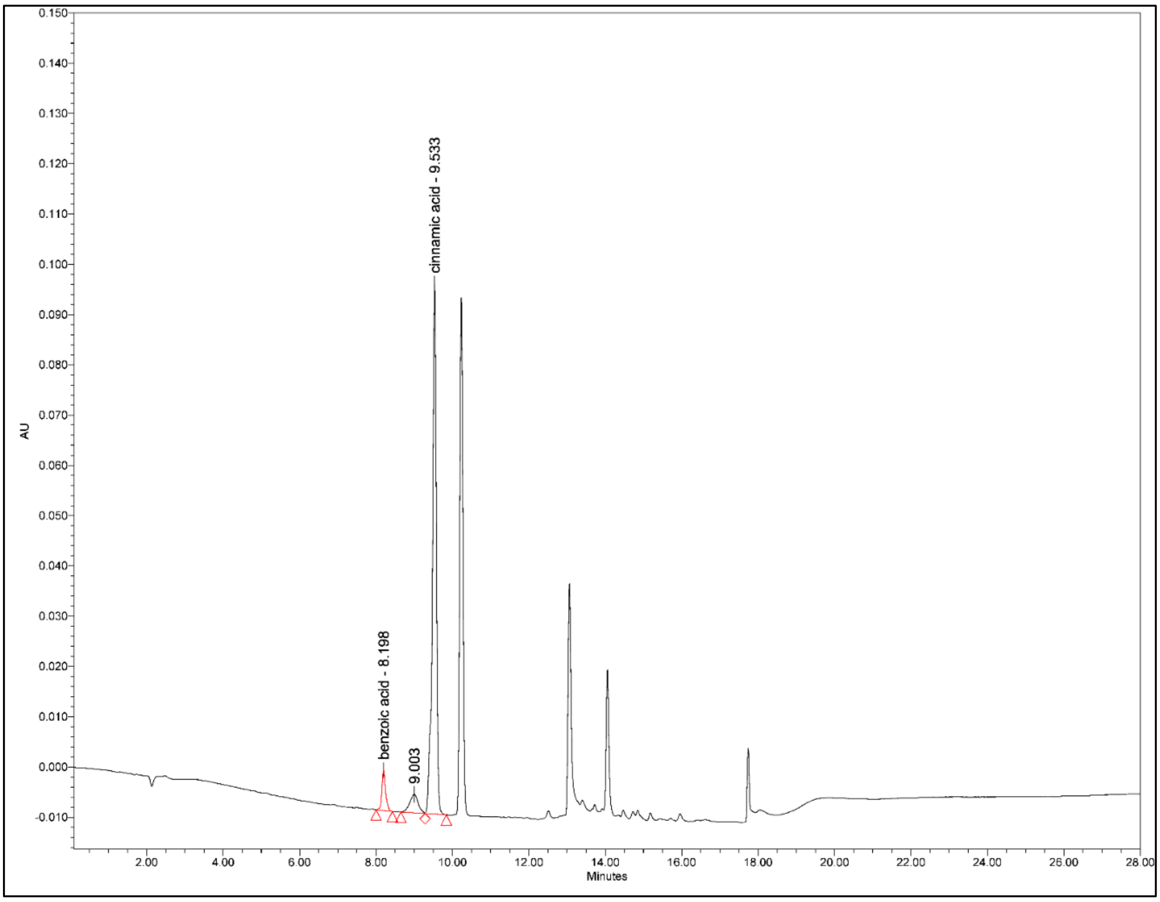

AU
Minutes
benzoic acid - 8.198
9.003
cinnamic acid - 9.533

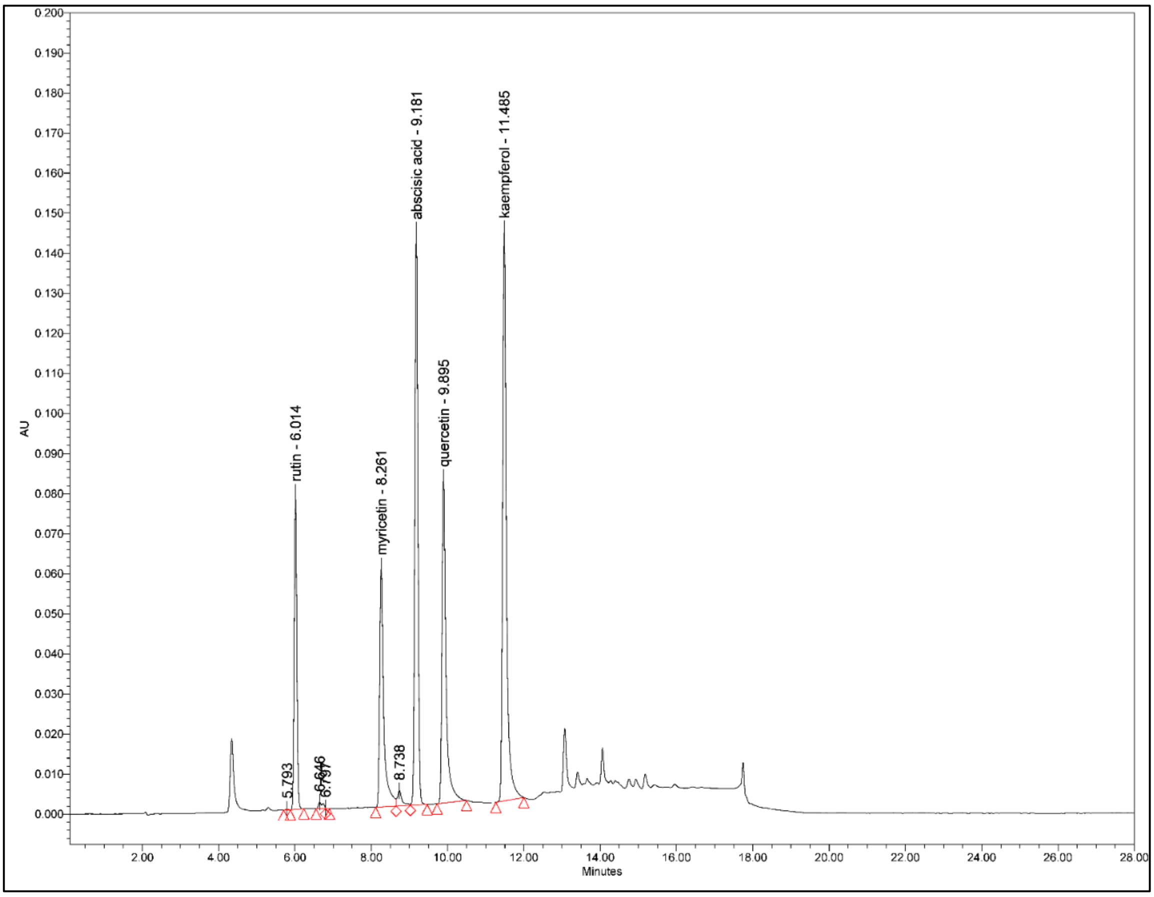

AU
Minutes
rutin - 6.014
5.793
6.646
6.797
myricetin - 8.261
8.738
abscisic acid - 9.181
quercetin - 9.895
kaempferol - 11.485

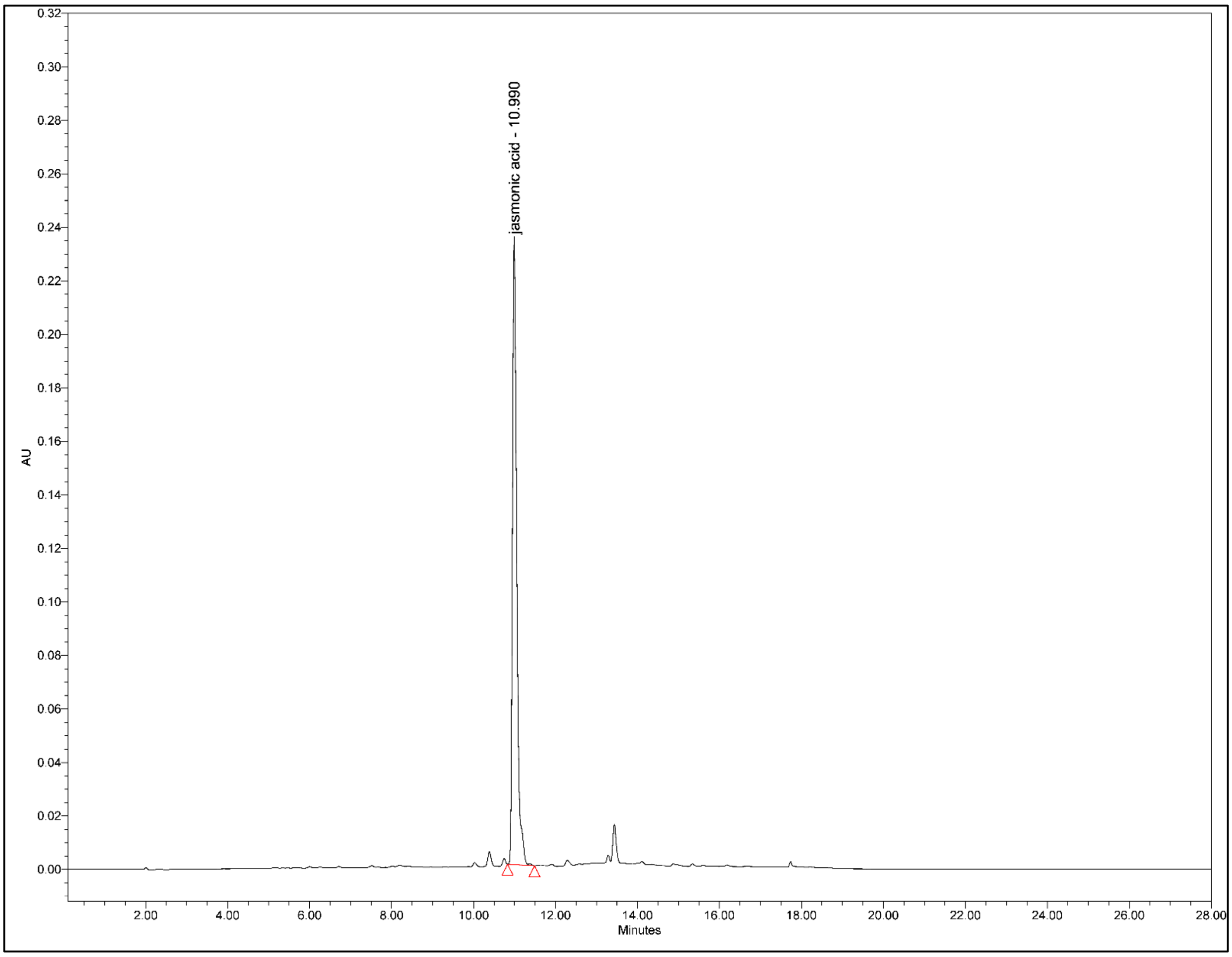

W474 scanning fluorescence detector (Waters):

Salicylic acid: excitation$_{\lambda}$: 305 nm; emission$_{\lambda}$: 407 nm; RT: 9.392

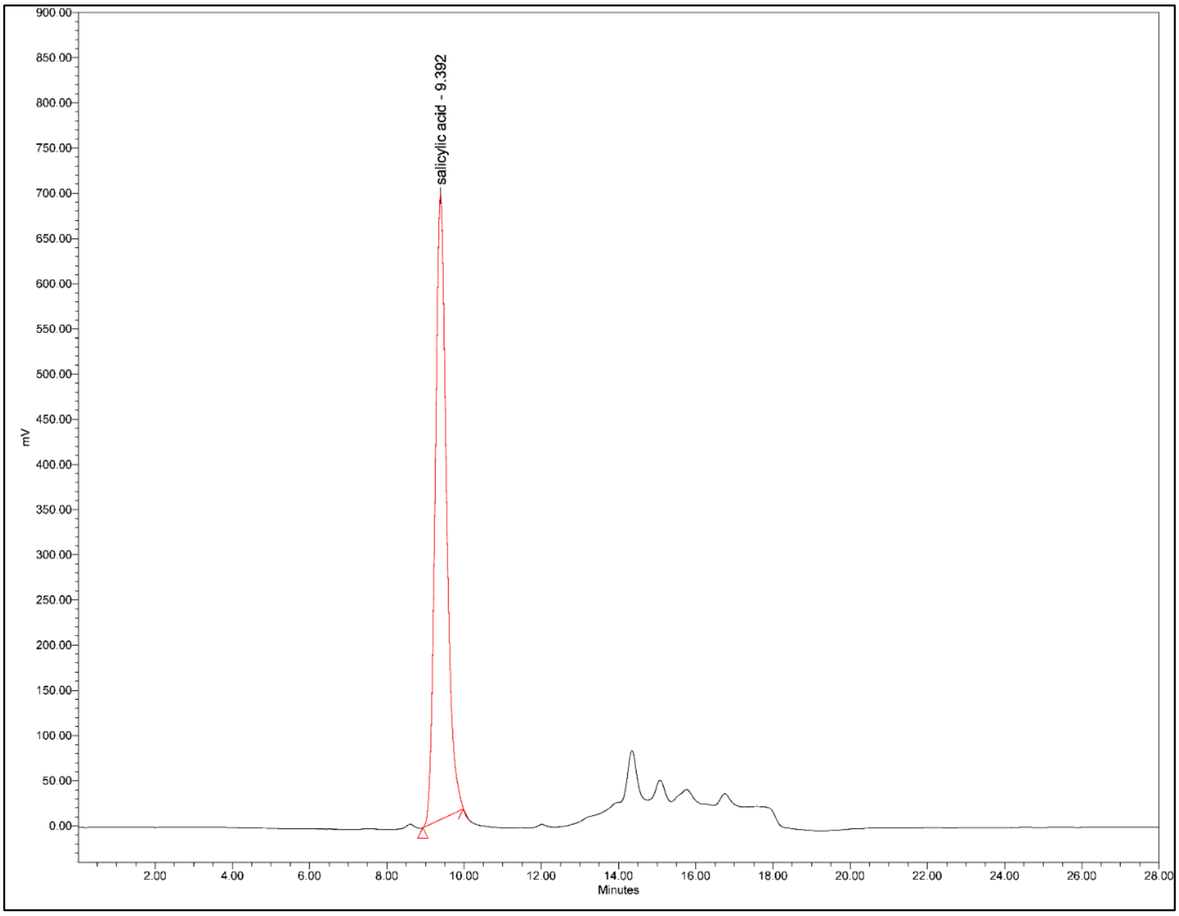

B) Glutathione

W2690 separation modul (Waters)

Column: HyperPrep HS C18 100A 8 µm 250x4.6 mm (ThermoScientific)

Pre-column: HyperPrep HS BDS C18 100A 8 µm 10x4 mm (ThermoScientific)

Column temperature: 30 °C

Sample temperature: 10 °C

Solvents:

A: 10% methanol+0.25% CH_3COOH pH 3.9

B: 90% methanol+0.25% CH_3COOH

Flow rate: 1 ml min^{-1}

Gradient:

Time	A%	B%
Initial	100	0
10	92	8
15	86	14
17.5	0	100
25.5	0	100
26.5	100	0
31	100	0

Detector parameters:

W474 scanning fluorescence detector (Waters):

$excitation_\lambda$: 380 nm; $emission_\lambda$: 480 nm; RT: 16.153

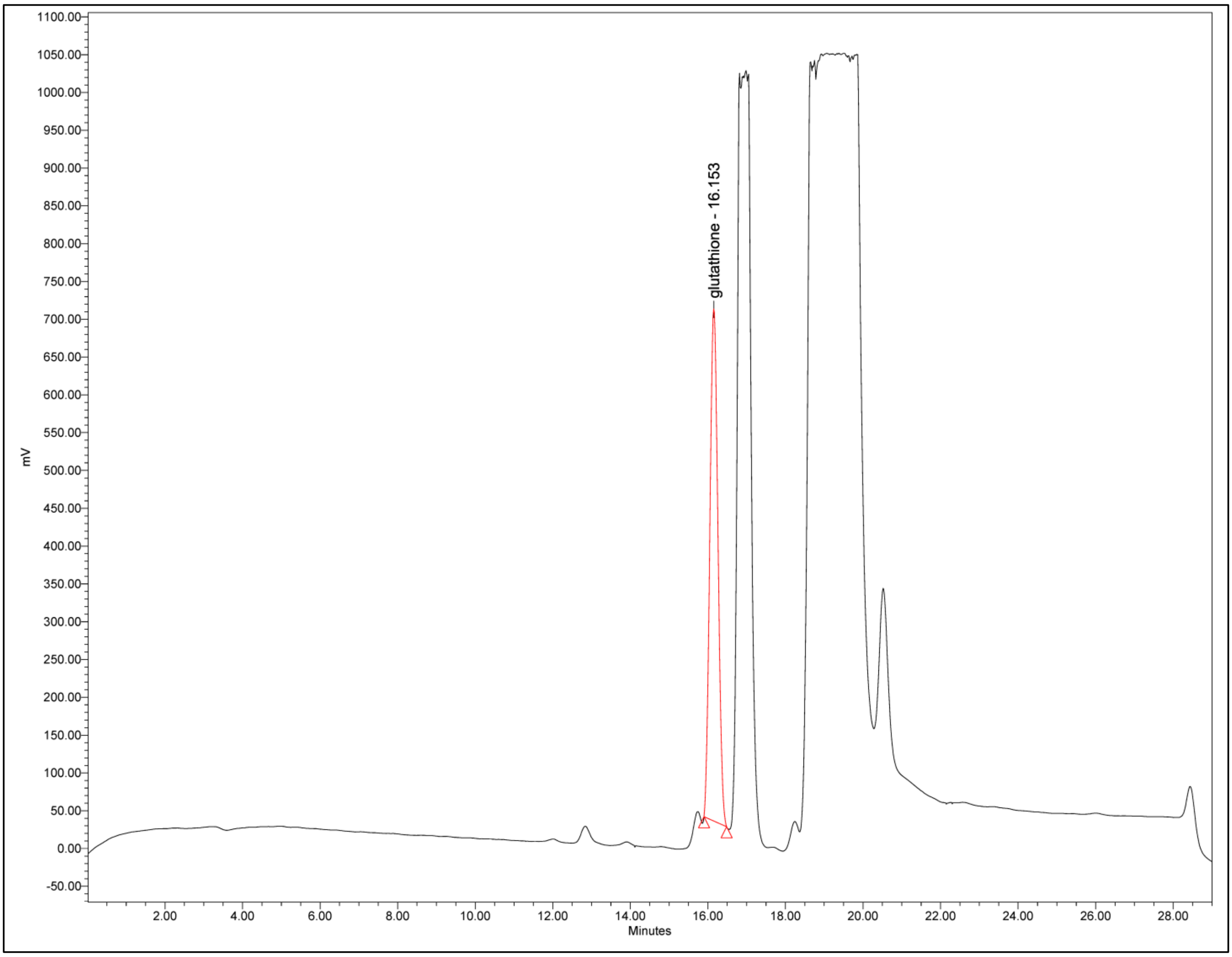
glutathione - 16.153
mV
Minutes
1100.00
1050.00
1000.00
950.00
900.00
850.00
800.00
750.00
700.00
650.00
600.00
550.00
500.00
450.00
400.00
350.00
300.00
250.00
200.00
150.00
100.00
50.00
0.00
-50.00
2.00
4.00
6.00
8.00
10.00
12.00
14.00
16.00
18.00
20.00
22.00
24.00
26.00
28.00
